# Study protocol: an evaluation of the effectiveness, experiences and costs of a patient-directed strategy compared with a multi-faceted strategy to implement physical cancer rehabilitation programmes for cancer survivors in a European healthcare system; a controlled before and after study

**DOI:** 10.1186/s13012-015-0312-3

**Published:** 2015-09-07

**Authors:** Charlotte IJsbrandy, Petronella B. Ottevanger, Wim G. Groen, Winald R. Gerritsen, Wim H. van Harten, Rosella P. M. G. Hermens

**Affiliations:** Scientific Institute for Quality of Healthcare (IQ healthcare), Radboud University Medical Centre Nijmegen, PO box 9101, 6500 HB Nijmegen, The Netherlands; Department of Medical Oncology, Radboud University Medical Centre Nijmegen, PO box 9101, 6500 HB Nijmegen, The Netherlands; Netherlands Cancer Institute, Division of Psychosocial Research and Epidemiology, Plesmanlaan 121, 1066 CX Amsterdam, The Netherlands; Department of Health Technology and Services Research, MB-HTSR, University of Twente, PO Box 217, 7500 AE Enschede, The Netherlands

## Abstract

**Background:**

The need for physical cancer rehabilitation programmes (PCRPs), addressing adverse effects from cancer, is growing. Implementing these programmes into daily practice is still a challenge.

Since barriers for successful implementation often arise at different levels in healthcare, multi-faceted strategies focusing on multiple levels are likely more effective than single-faceted strategies. Nevertheless, most studies implementing PCRPs used strategies directed at patients only. The aim of this study is to develop and identify the most effective strategy to implement PCRPs into daily care. We want to assess the added value of a multi-faceted strategy compared with a single-faceted patient-directed strategy.

**Methods/design:**

We will conduct a clustered controlled before and after study (CBA) in the Netherlands that compares two strategies to implement PCRPs. The patient-directed (PD) strategy (five hospitals) will focus on change at the patient level. The multi-faceted (MF) strategy (five hospitals) will focus on change at the patient, professional and organizational levels. Eligibility criteria are as follows: (A) patients: adults; preferably (history of) cancer in the gastro-intestinal, reproductive and/or urological system; successful primary treatment; and without recurrence/metastases. (B) Healthcare professionals: involved in cancer care.

A stepwise approach will be followed:

- Step 1: Analysis of the current implementation of PCRPs and the examination of barriers and facilitators for implementation, via a qualitative study with patients (four focus groups *n* = 10–12) and their healthcare workers (four focus groups *n* = 10–12 and individual interviews *n* = 30–40) and collecting data on adherence to quality indicators (*n* = 500 patients, 50 per hospital).
- Step 2: Selection and development of interventions to create a PD and MF strategy during expert roundtable discussions, using the knowledge gained in step 1 and a literature search of the effect of strategies for implementing PCRPs.
- Step 3: Test and compare both strategies with a clustered CBA (effectiveness, process evaluation and costs), by data extraction from existing registration systems, questionnaires and interviews. For the effectiveness and cost-effectiveness, *n* = 500 patients, 50 per hospital. For the process evaluation, *n* = 50 patients, 5 per hospital, and *n* = 40 healthcare professionals, 4 per hospital. Main outcome measures: % screened patients, % referrals to PCRPs, incremental costs and incremental cost-effectiveness ratios (ICERs).

**Trail registration:**

NCT02205853 (ClinicalTrials.gov)

## Background

The increased incidence of cancer, due to ageing and lifestyle of the western population and the increased survival rate, results in a large group of cancer survivors [1–6]. These patients face unique challenges from persistent adverse effects from their cancer and its treatment [7], which need to be addressed. Cancer and its treatment concerns loss of quality of life (QoL), cancer-related fatigue [8, 9] and other symptoms, such as pain, lymphoedema [10], insomnia and hormone and immune system dysfunctioning [11]. Symptoms depend on the type of cancer, type of treatment and characteristics of the patient and usually emerge before and during treatment and may remain even long after the completion of treatment [12–16]. The prevalence of cancer survivors, who experiencing symptoms of fatigue, is estimated around 20–40 % [8, 17–22]. A lower QoL is experienced by 35 % of the cancer survivors as well [17]. In addition, cancer survivors often have a marked declined cardiopulmonary fitness after finishing their primary treatment [18].

In view of the enormous increase in the number of cancer survivors, it is important to find ways to prevent or mitigate these physical and psychosocial symptoms. In recent years, the positive impact of physical cancer rehabilitation programmes (PCRPs) to counteract both physiologic and psychosocial adverse symptoms in cancer survivors was published [19–28]. Participation in PCRPs has shown to result in decreased fatigue [19, 29–38] and improved cardiopulmonary fitness [29] and QoL [29, 32, 34, 39–44]. Additionally, improvement of muscular strength [45], lean body mass, body fat levels [46], self-esteem and even better chemotherapy completion rates have been reported [47].

PCRPs can be offered in multiple ways (variation in intensity, frequency, duration and format) and at different time points in the cancer trajectory [48]. Regardless of the type of programme, cancer survivors should be stimulated to avoid inactivity and return to normal physical daily activities as soon as possible after surgery, and during adjuvant therapy, related to the WHO health-related physical activity guidelines for the general population [49].

Many experts have concluded that PCRPs are safe during and after cancer (treatment)s, resulting in improvement of many symptoms in cancer survivor groups and that they are probably appropriate to the need of cancer survivors [29, 48, 50–54]. Questionnaires that were rating the experiences of patients with the cancer care in Dutch hospitals showed that the experiences with receiving information and assistance concerning rehabilitation were overall negative. It also showed that patients define information and assistance for referral concerning rehabilitation important aspects of good quality cancer care [55, 56]. Additionally, patients define the information and assistance for referral concerning rehabilitation most suitable for improvement of cancer care in the Dutch healthcare. However, it appears that the uptake of PCRPs is rather low [57–60] and the implementation into daily practice still seems to be a challenge, due to several barriers situated at the patient, provider (hospital) and healthcare system levels [61]. In our experiences, lack of knowledge about effective implementation strategies, but mainly their cost-effectiveness and cost benefits, is a huge barrier on the level of the provider and healthcare systems. On the other hand, competition between providers gives hospitals enough reason to use cost-effective implementation strategies to improve the healthcare service experiences of their patients. A study evaluating the most effective implementation strategy, showing also which aspects of these strategies generate more positive experiences and show cost-effectiveness, might be a catalyst for the implementation of PCRPs.

Studies on implementation of PCRPs are scarce [62–67], but in the last 15 years, over 70 papers on implementing research findings have shed light on the effectiveness of implementation strategies in different aspects of healthcare [68–71]. Various strategies are advocated for the implementation of healthcare innovations, each based on different assumptions and theories on human behaviour and organizations [72–74]. Most theories emphasize that effective implementation cannot take place without a systematic approach and appropriate preparation and planning [75, 76]. Knowledge gained from implementation studies in different aspects of healthcare shows that a tailored implementation strategy should be designed according to the specific features of the innovation, the target group, the setting and the barriers to change. After this, the designed implementation strategy can be tested on effectiveness, feasibility and costs [77, 78] and if needed readjusted.

Since barriers often arise at different levels in the healthcare system, it is very likely that a multi-faceted strategy focusing on different levels (patient, professional and/or the organizational level of care) is more effective than single-faceted strategies [78–81]. Nevertheless, most studies published use single-faceted strategies directed mainly at patients to support the success of implementing PCRPs. Common used patient-directed strategies are patient-empowerment-enhancing tools, often delivered by information and communication technology (ICT). Examples are the use of a website for (1) distribution of educational materials, (2) patient-oriented theoretical feedback based on questionnaires, (3) reminders via e-mail and social media platforms [82] and (4) stimulating monitoring health systems containing heart rate monitors and pedometers [83]. In the process of rehabilitation, empowerment-enhancing tools can have extra value, since confidence to take charge, make decisions and belief in yourself can directly affect the efficacy of the rehabilitation [84]. Sufficient empowerment gives individuals the capacity to influence the behaviour of themselves and others, like the professionals involved in their care [85].

However, strategies aimed at professionals (educational outreach, audit and feedback) can also result in successful implementation (professional performance, patient outcomes and costs) [86, 87]. Strategies directed on the organization could support the implementation of PCRPs as well, mainly because contextual factors, such as workload, poor coordination and management, can be important barriers for success.

In our present study, we aim to identify the most effective strategy to implement PCRPs into daily care. Specific goals are to (1) gain insight into the specific features needed for a successful implementation by exploring the setting and factors (barriers and facilitators) that might influence the implementation of PCRPs. After this, (2) we aim to develop a patient-directed strategy and a multi-faceted strategy. The patient-directed strategy will be designed to embed the success of implementation of PCRPs by influencing the patients, and the multi-faceted strategy will be designed to embed the success by not only influencing the patients but also professionals and organizational aspects. Both strategies will be designed tailored to the setting and found factors affecting implementation. We also aim to (3) compare the effectiveness, experiences of participants (process evaluation) and costs of both strategies on the hospital and patient level and (4) get insight into the additional effects of strategies directed also at professionals and organization aspects compared to patient-only strategies.

Our first hypothesis is that hospitals with a multi-faceted strategy will have a more effective implementation of PCRPs in comparison with hospitals with a patient-directed strategy.

Our second hypothesis is that the patients and professionals will experience more positive experiences with the multi-faceted strategy in comparison with hospitals with a patient-directed strategy.

Our third hypothesis is that those hospitals with the multi-faceted strategy will have higher costs (incremental costs) in comparison with hospitals using the patient-directed strategy.

Our last hypothesis is that the incremental cost-effectiveness ratio (ICER) of the multi-faceted strategy will be lower than the patient-directed strategy.

## Methods/design

### Setting

This study is part of the Alpe d’HuZes Cancer Rehabilitation (A-CaRe) programme, which includes multiple projects with two main purposes (1) to evaluate the (cost-) effectiveness of rehabilitation programmes for cancer patients, A-CaRe 1 [88–95], and (2) to implement these programmes in daily patient care, A-Care 2 [96–100]. Our study concerns the A-Care 2 programme. The Committee on Research Involving Human Subjects of the region Arnhem-Nijmegen of the Netherlands assessed the study and concluded that our study will be carried out in accordance with the applicable rules concerning the review of research ethics committees and informed consent.

### Design

This study is a clustered controlled before and after study (CBA).

It will compare a patient-directed strategy with a multi-faceted strategy to implement evidenced-based PCRPs. We consider PCRPs evidence based, when the features of the programme are in line with the recommendations of the evidence-based Dutch guidelines (‘Cancer Rehabilitation’ , and ‘Cancer Survivorship Care’) and additional international literature and guidelines [19, 22, 23, 29, 101].

**Fig. 1.**
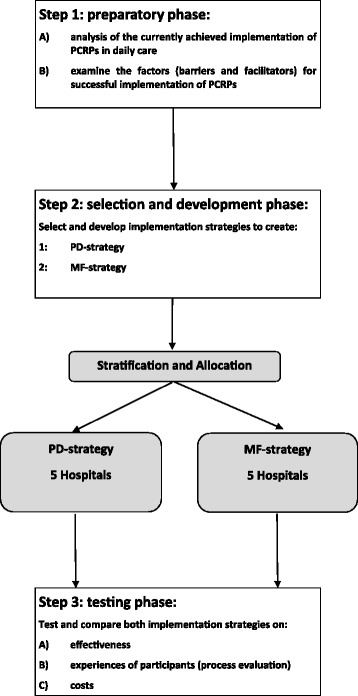
Overview of the planned stepwise approach of the study

For the selection, development and testing of the implementation strategies, we will follow a stepwise and structured approach, summarized in Fig. 1, based on the Grol and Wensing implementation of change model [81].

In *step 1*, we will first analyse the currently achieved implementation of PCRPs in daily care and examine the factors (barriers and facilitators) that might influence optimal implementation of PCRPs. In *step 2*, we will use the background information of step 1 and a literature study to develop and tailor implementation strategies to the target group, the setting and the barriers to change. In *step 3*, we will then compare newly achieved implementation with the implementation measurements from step 1 to assess and compare the effectiveness of the two implementation strategies by a CBA design. We will also conduct a process evaluation and cost analysis. Clustering of the CBA study will be on the hospital level as matched pairs in a 1:1 ratio. The hospitals are situated in different regions in the Netherlands, and hospitals will be stratified by the study group, according to type of hospital (university, teaching and non-teaching hospitals) and capacity (number of beds).

Each group will contain one university, two teaching and two non-teaching hospitals in the Netherlands.

During our study, healthcare professionals of the participating hospitals will hand out patient information and informed consent to all eligible patients. Patients that give informed consent will be approached, screened and if eligible included as participant for one or more analyses of our study.

Professionals are asked for participation by writing. Professionals that give informed consent will be screened and if eligible included as a professional participant for one or more analyses of our study.

### Step 1: preparatory phase

#### Analysis of the currently achieved implementation of PCRPs in daily care

Quality indicators will be used to explore the actual implementation of the PCRPs.

##### Study population

Structure indicators will be collected from the 10 participating hospitals, and process and outcome indicator data will be collected from 500 adult cancer patients in the participating hospitals (about 50 adult patients per hospital). The group of patients included are patients with preferably (a history of) cancer located in the gastro-intestinal, reproductive and/or urological systems and that have successfully passed their primary treatment without signs of recurrence or metastases.

##### Data collection

The quality indicators will be developed by a panel of 10–12 professional experts and patients, using the RAND-modified Delphi method [102, 103]. These indicators will be developed to measure the success of implementation and adherence to the recommendations of the Dutch guideline ‘Cancer Rehabilitation’ . With these extracted quality indicators (structure, process and outcome) and frequently used outcome indicators as QoL and patient empowerment, actual performance of the current implementation of PCRPs will be measured in the participating 10 hospitals. Data about adherence to the structure quality indicators will be collected consulting a panel consisting of healthcare professionals, management and patients from the 10 participating hospitals. Data about adherence to the processes and outcome quality indicators will be retrieved retrospectively from existing registration systems. Data not available will be surveyed by questionnaires. For example, to measure the outcome indicators QoL and patient empowerment, we will use the European Organisation for Research and Treatment of Cancer Quality of life Questionnaire-C30 (QLQ-C30) and the Patient Activity Measure (PAM) questionnaire.

We will also collect data about patient characteristics such as age, cancer type, clinical stage, co-morbidity, performance status and types of therapy to find possible confounding factors.

All indicators will be measured, using a data extraction form. A data extraction form will also be developed and pilot tested to register patient characteristics and other aspects relevant to assess the extent of implementation.

##### Planned analytic approach and outcomes

The main outcome measure is the adherence to quality indicators (process and structure indicators) from the PCRPs and their implementation (for example, outcomes of the QLQ-C30 and the PAM questionnaires).

In this measurement, adherence to the quality indicators will be used as dependent variables and patient characteristics (e.g. age, cancer type, clinical stage, co-morbidity, performance status and types of therapy) will be included as possible confounders.

#### Examine the factors (barriers and facilitators) for successful implementation of PCRPs

The qualitative study will be based on focus-group interviews and individual interviews.

##### Study population

The participants invited for the focus groups and individual interviews will be recruited from the 10 hospitals that are participating in our study.

The focus-group interviews concern the following groups: (a) four groups of 10–12 adult patients with preferably (a history of) cancer located in the gastro-intestinal, reproductive and/or urological systems, receiving treatment with curative intent or that have successfully passed their primary treatment. (b) Four groups of 10–12 professionals involved in the treatment of these cancer patients in secondary care (e.g. surgeons, radiotherapists, medical oncologists, gynaecologist, urologist, rehabilitation physicians, sports-medicine physicians, physical therapists, physician assistants and psychologists).

Additional to the focus-group interviews, we will conduct individual interviews with 30–40 professionals involved in the treatment of cancer patients in primary care (e.g. general practitioners, physical therapists and psychologists).

##### Data collection

The outcomes of the qualitative study will be collected through a semi-structured interview process. Four interview guides (one for patients, one for professionals involved in the secondary care, one for general practitioners and one for physical therapists and psychologists) will be developed using theoretical models developed by Cabana [104] and Grol [77] for identification of influencing factors. These models have four similar domains: characteristics of the innovation itself, characteristics of professionals, characteristics of patients and characteristics of the organization and context in which the innovation has to be applied. All interviews will be audio recorded and verbatim written in a document.

Besides the factors collected during the interviews, we will collect the characteristics of the participants of the interviews. Patients are asked to fill in a form that states their age, sex, home setting, working situation, cancer type and types of therapy. Professionals are asked to fill in a form that states their age, sex, years of practice, function and speciality.

##### Planned analytic approach and outcomes

The barriers and facilitators mentioned in the interviews will be independently qualitatively analysed by two researchers, using the qualitative software package Atlas.ti version 7 and will be descriptive. The factors identified will be classified within the framework that has been developed by Cabana [104] and Grol [77]. Factors identified, but not present in the models, will be added. The two sets of scores from the two researchers will be compared, and any discrepancies will be discussed until consensus is achieved. This approach will provide barriers and facilitators on different levels, including features of the PCRPs, the group of professionals involved in the care, group of patients and organization and context in which the PCRPs have to be applied.

### Step 2: selection and development phase

We will select and develop two implementation strategies to implement the PCRPs. We will create:

1. A single-faceted patient-directed (PD) strategy that will embed the change at the patient level.
2. A multi-faceted (MF) strategy that will embed the change at the patient, professional and organizational levels. It will also consist of the same patient-directed elements as the PD strategy.

Hospitals are requested for participation in our study by an invitation letter that will be sent by e-mail or post, and after agreement, they will receive full information for participation through a presentation. Interested hospitals will then meet with the study personnel (e.g. project manager, senior researcher and junior research fellow) in order to reach a clear understanding of all study components and to provide written informed consent. After inclusion, the hospitals will be allocated to either the PD- or MF-strategy group, taking into account sufficiently comparable settings and organizations in both the PD- and MF-strategy groups. In the Netherlands, cancers of the gastro-intestinal, reproductive and urological systems account for more than 35 % of the incidence of all cancers, being more than twice the incidence of breast cancer. Until now, however, most cancer rehabilitation programmes are designed and focused on breast cancer. Patients with gastro-intestinal and gynaecological cancers judge their cancer care low in comparison with patients with other tumour types. [55]. Patients with gastro-intestinal cancers also rated the information after finishing the primary treatment significantly lower in comparison with breast cancer patients [105]. Therefore, we choose to focus on cancer patients and their healthcare professionals in the care pathways for gastro-intestinal, gynaecological and urological oncology.

To develop the PD and MF strategies to deploy cancer rehabilitation programme(s), a literature search will be done. This literature search will be performed in MEDLINE, Embase and CINAHL, to define the evidence of implementation strategies for PCRPs. The search will be limited to studies of human beings and will have an English language restriction. The search terms will include the methodological filters of the 'Cochrane EPOC (Effective Practice and Organisation of Care) group' combined with selected MeSH terms and free-text terms. We will also use the snowball method for inclusion of further relevant trials.

Inclusion criteria are as follows:

1. Population:
  - Adult cancer patients and
  - Healthcare workers involved in their care

We will not include studies in non-adult populations because of the differences in medical decision-making regarding children and adolescents, including the parents/guardian role.

2. Intervention:

Strategies aimed at improving exercise during and after cancer. We define implementation as any planned process and systematic introduction of guidelines, healthcare innovations or health behaviour, aiming to be given a structural place in the patient’s life and professional practice.

We consider any of the strategies detailed in section 2 ‘Interventions’ of the 'EPOC Data Collection Checklist 2002':

- Professional interventions
- Financial interventions
- Organizational interventions
- Regulatory interventions

In the 'EPOC Data Collection Checklist 2002', interventions focused on patients are categorized under financial and organizational interventions. Since we believe that nowadays interventions focused on patients are an important part of implementation strategies, we decided to add the category patient interventions to the list of ‘Interventions’ . We decided to use the following subjections under the category patient interventions, related to the subjection of the category professional interventions:

- Distribution of educational materials
- Educational meetings
- Local consensus processes
- Educational outreach visits
- Local opinion leaders (in this category, healthcare professionals)
- Patient-mediated interventions
- Audit and feedback
- Reminders
- Marketing and
- Mass media

We will include any active or passive implementation strategy. The combination of two or more strategies will also be considered as a valid intervention. Both studies describing implementation strategies alone or additionally to PCRPs will be included.

3. Comparison:
  - Outcomes before the introduction of the rehabilitation programme or strategy or
  - Outcome comparison group
    i. Where another programme or
    ii. Control: no programme or usual care was implemented

4. Type of outcome:
  - All outcomes of ‘type of targeted behaviour’ section 2.3 of the 'EPOC Data Collection Checklist 2002'

The results of the literature search will, together with the knowledge based on the results of the preparatory phase in step 1, be the basis to select and develop the implementation strategies during roundtable discussions. For the five hospitals of the PD-strategy group, a single-faceted patient-directed strategy will be designed that will embed the change only at the patient level. For the hospitals of the MF-strategy group, a multi-faceted strategy will be designed that will embed the change at the patient, professional and organizational levels. The MF strategy will consist also of the PD strategy, so the additional value of the strategies directed at the professional and organization can be evaluated afterwards. For the roundtable discussions, an expert panel will be composed consisting of representatives from the participating hospitals (patients, healthcare professionals and hospital managers), experts on cancer rehabilitation and experts on implementation science.

### Step 3: testing phase

The designed implementation strategies will be used to deploy the PCRPs in daily care and will be evaluated and compared on (A) effectiveness, (B) experiences of participants (process evaluation) and (C) costs. In all participating hospitals, the trial will be conducted for a half to 1 year, depending on the number of cancer patients per year in the participating hospitals allowing for fast accrual. Between 6 to 12 months after introduction of the implementation strategies, the outcomes of the PD and MF strategies will be evaluated and compared.

#### To test and compare both implementation strategies in terms of effectiveness

##### Study population

The study population in this evaluation will have the same characteristics as the study population in the ‘Analysis of the currently achieved implementation of PCRPs in daily care’ section.

##### Data collection

As described in the ‘Analysis of the currently achieved implementation of PCRPs in daily care’ section, quality indicators will be used to explore the actual achieved implementation of the PCRPs after the introduction of the developed implementation strategies. By comparing the implementation of PCRPs before and during the implementation period, we can evaluate the effectiveness of the strategies. The process of data collection of the quality indicators during the implementation period will be similar as the pre-measurement described in the ‘Analysis of the currently achieved implementation of PCRPs in daily care’ section.

##### Study outcomes

The primary outcome measures are two quality indicators: (A) the percentage of screened patients with the screening tool recommended by the Dutch guideline ‘Cancer Rehabilitation’ and (B) the percentage of referrals to PCRPs where needed, according to the Dutch guideline ‘Cancer Rehabilitation’.

Secondary outcome measures are the differences in adherence between the pre- and post-measurement to the remaining quality indicators (process and structure indicators) extracted from the Dutch guideline ‘Cancer Rehabilitation’ and often-used outcome indicators as QoL and patient empowerment.

##### Power calculation

A priori sample size calculations were based on the primary outcome measures, percentage of screened patients and percentage of referrals to PCRPs where needed. A power analysis for a comparison between the PD- and MF-strategy groups on the primary outcomes estimated a sample size of five hospitals per group (*n* = 50 patients per hospital) with a power of 0.80 and a two-tailed alpha of 0.05 and accounting for an estimated dropout of 10 %. Specifically, to detect an effect size of 50 % in the MF-strategy group and of 20 % in the PD-strategy group.

##### Planned analytic approach

Both univariate and multivariate (multi-level) analyses will be performed to obtain an indication of the effectiveness of the implementation strategies in increasing the proportion of patients who are treated according to the programme before and after applying the implementation strategy. In this analysis, adherence to the quality indicators will be used as dependent variables and patient characteristics (e.g. age, cancer type, clinical stage, co-morbidity, performance status and types of therapy) will be included as possible confounders. The results of the ‘post-measurement’ will be compared with the ‘pre-measurement’ . Additionally, the differences of the pre-post-measurements will be compared between the PD- and MF-strategy groups.

If possible, all outcomes (effectiveness, experiences of participants (process evaluation) and costs) will be analysed at the patient level (all patients of hospital PD strategy vs. all patients of hospital MF strategy). Additionally, we will analyse at the hospital level to explore the effects of the strategies related to hospital characteristics (university, teaching and non-teaching hospitals).

#### To test and compare both implementation strategies in terms of experiences of participants (process evaluation)

##### Study population

For the process evaluation, a sample of adult patients (*N* = 50; 5 per hospital) and healthcare professionals involved in their cancer care (*N* = 40; 4 per hospital) will be included. The patients included are again adult patients, diagnosed with cancer in the abdominal or pelvic cavity (ea. gastrointestinal, reproductive and urological system).

##### Data collection

In order to study the use of and experiences with the different elements of the implementation strategies and the modified care, a process evaluation will be performed. This will include the extent to which patients and professionals (when the strategy includes patient- and professional- directed elements) used the elements of the strategies and their experiences (e.g. satisfaction and feasibility) with these elements and the modified care. Process information will be gathered in a qualitative study in the 10 hospitals, in which the implementation strategies were tested. Individual interviews will take place among the patients and hospital professionals. For example, if a website for cancer patients with information about the importance of physical exercise is part of the strategy, patients are asked about the frequency they visited this website, their satisfaction with this website and how feasible it is to use the website. All interviews will be recorded and typed in a document.

##### Study outcomes

The use of and experiences with the elements of the strategies and the modified care by patients and professionals will be studied (both participation in and satisfaction with the different elements).

##### Planned analytic approach

For the process evaluation, interviews with professionals and adult cancer patients will be qualitatively analysed, using the qualitative software package Atlas.ti version 7. The process analysis will be descriptive. The descriptive outcomes of the PD- and MF-strategy group will be compared.

#### To test and compare both implementation strategies in terms of costs

To determine the cost and benefits per patient of the implementation strategies and their consequences, we will use an activity-based costing (ABC) approach [106]. ABC was developed by Robert S. Kaplan in the mid-1980s [107]. Academic researchers and accounting practitioners believe it is the most modern normative appropriate costing system that can properly and confidently give information for decision-making. ABC helps to get a more realistic view of the indirect costs by using multiple cost drivers. In ABC, the indirect cost is assigned to activities and products (healthcare services), via a cause and effect relationship. Healthcare systems are considered as an entity that includes series of activities for the purpose of performing healthcare services to the patients. These activities in a healthcare system generate costs, which include direct and indirect costs, but also benefits. Next to accurate cost information, ABC also provides a clear view of the activities in the healthcare services that might be used for evaluating healthcare process performances.

##### Study population

For the cost evaluation, the 10 hospitals of our study, their patients and professionals will be included. For the outline of the patient care processes, we will use a sample of 500 patients (about 50 adult patients per hospital). The characteristics of the patients will be the same as the characteristics of the study group of the ‘Analysis of the currently achieved implementation of PCRPs in daily care’ section. The meetings with professionals from the participating hospitals to reach a consensus on the protocol of processes (activities) of the PD and MF strategies will consist of 20 professionals, 10 professionals per cluster. All professionals should be involved in the treatment of cancer patients and the implementation processes of the PCRPs.

##### Data collection and planned analytic approach

By an ABC approach, we will try to calculate the actual costs and benefits for using the PD- and MF-implementation strategies. In order to accurately assign the costs to the implementation strategies and followed healthcare services, it is necessary to determine exactly the consumption of activities. Afterwards, we need to assign the costs of resources needed to deliver these activities.

The activities and the followed costs of resources (personnel, material and amortization of investment costs) to implement the PCRPs via the PD- or MF-implementation strategies are perceived as the costs, according to ABC approach. On other hand, the prevented activities and their costs of resources that are not used due the implementation of the PCRPs by the PD- or MF-implementation strategies are considered as the benefits. The costs will be measured from the time of preparation and development of strategies until 1 year after the start of the implementation strategies.

Our first step will be to develop protocols of activities performed during the (1) preparation and development of the strategies and (2) activities accumulated by the activity of the healthcare implementation processes (processes of care of patients involved in the implementation processes).

To determine the activities, we will select a sample of patients involved in the implementation process and outline the patient care processes (activities) in each case.

During meetings with professionals from the participating hospitals, we will try to reach a consensus on the protocol of processes (activities) of the PD and MF strategies. The protocol of processes will outline a description of processes and details of their constituent activities.

Our next step will be to proceed the allocation of resources used in each process. For collecting information on the use of the different elements of the implementation plan by professionals and patients, existing databases will be used as much as possible. When necessary, registration forms will be developed and will be completed by the professionals and the sample of patients involved in the implementation process. The input of resources in the implementation strategy will be assessed by collecting volumes of use of the different elements of the implementation strategy by professionals and patients and multiplying these by the price of each element (market prices, guideline prices or self-determined prices based on costing methods, i.e. full costing). Future costs and effects will be discounted at 4 and 1.5 %, respectively, according to the Dutch guidelines [108].

Cost prices of the processes will be determined using standard unit cost prices according to the Dutch guidelines for costing research [108]. If certain standard unit cost prices are not available, real cost prices will be determined by consulting the management of participating hospitals.

With the protocol of processes of activities and their cost of resources, we will calculate the incremental costs of both the PD and MF strategies. We will also calculate an incremental cost-effectiveness ratio (ICER). The outcomes used for calculating the ICER are the primary outcome measures of our effectiveness study (the ‘To test and compare both implementation strategies in terms of effectiveness’ section): (1) percentage of screened patients and (2) percentage of referrals to PCRPs were needed.

After this, we will compare the expected incremental cost (and potential savings) and cost-effectiveness ratios of the PD with the MF strategy.

##### Study outcomes

The expected costs (and potential savings) and ICERs of the development and use of the PD and MF strategies. Additionally, we will compare the outcomes of the PD strategy with the MF strategy.

### Trial status

At the time of manuscript submission, the study is ongoing at step 1, the preparatory phase. Some interviews for examination of the factors (barriers and facilitators) for successful implementation of PCRPs have been performed, but no data analysis has begun.

Data on quality indicators, to analyse the actual performance of the current implementation of PCRPs in daily care, have not been gathered.

## Discussion

This is one of the few studies to develop and test two implementation strategies to implement PCRPs into daily healthcare in a structured and stepwise approach. In previous papers, PCRPs were implemented in German, United Kingdom, Canadian and American healthcare systems [62, 64–67]. To our knowledge, a study on the effectiveness and feasibility of implementation of PCRPs in a stepwise structured approach and performed on a large scale in the Dutch healthcare system has not been conducted before. The originality of our study is further supported by being one of the few that compares the effect of a multi-faceted strategy with a patient-directed strategy.

During the process of selecting strategies, it is important that the strategies will be evidence based and tailored to the barriers and facilitators that are directed on patients and in the MF-strategy group additionally also on professionals and the organization level. It is best to arrive to a cost-effective mix of measures and activities, which has preferably been found to be effective in similar situations [77].

Multi-faceted strategies targeting different barriers in different fields (patient, professional and organization) are more likely to be effective than single ones [71]. Therefore, it is more likely that our MF strategy, focusing also at professionals and the organization, will be more effective than the strategy that only embeds the change on the level of the patient. In most studies, the implementation of PCRPs is mainly supported by strategies directed at patients. Patient strategies, like empowerment-enhancing tools, are promising since they fit the patient-centred care approach and stimulate an active patient role and have the potential to improve the rehabilitation process itself [109, 110]. Enhancing empowerment has shown its clinical effect in the rehabilitation of patients with osteoporosis [111], hip fracture [112], and might also have its effect on the success of implementing PCRPs. Promising strategies directed at professionals are mainly active strategies, including educational outreach visits, reminders and audit. And a promising strategy directed at organizations is enhancing professional roles, healthcare pathways and mainly tools in an ICT format [86]. Experts conclude that further research is required to discover which strategies to present to target the different barriers and settings [71, 79].

With regard to the implementation of PCRPs, it is possible that strategies in an ICT format can contribute to the effectiveness of the implementation [96, 98, 113]. Implementation experts consider ICT to be the key to improve efficiency and quality of healthcare [114]. In our opinion, ICT can be effective at the level of the professionals or the healthcare teams to improve adherence to the programmes by clinical decision support systems, surveillance and monitoring. At the level of the patients, it can realize new forms of healthcare services outside the healthcare institutions and in the patient’s environment instead. It can also increase the level of patient empowerment by giving the opportunity to get insight in his/her medical data and become more involved in the rehabilitation process [115]. Additionally, ICT may also support the physical rehabilitation process by restoring health and improving outcomes, since previous studies have shown that ICT interventions may lead to improved physical activity levels, with promising outcomes in several patient groups [96, 116–119]. ICT tools and tools to enhance patient empowerment, designed and piloted by other A-CaRe research groups, will be incorporated in our implementation strategies.

Healthcare improvement programmes are well-known to be better adopted by socio-economically advantaged individuals as compared to disadvantaged individuals [120, 121]. Moreover, younger individuals are able to use and access ICT innovations easier than older individuals [122]. The design of our strategies will be developed with a pragmatic attitude [123, 124] to avoid increasing health inequalities by providing equal access to the PCRPs. Patients will not be selected on their exercise ability or history, and criteria are not restrictive to a particular population to fit with real clinical context. Nevertheless, we will focus on adult patient populations and will not include non-adult populations because of the differences in medical decision-making regarding children and adolescents, including the parent/guardian role and the different tumour types.

An important limitation of our study is the absence of a proper randomized study design. This would eliminate bias in implementation strategy assignment and improve the evidence that differences in outcomes between the implementation strategies indicate significant effects on physical cancer rehabilitation care [125]. It is, however, also known that comparing complex interventions and programmes is a challenging matter, for which one has to settle with less advanced designs.

Our implementation strategies have the disadvantage of measuring the effect of the entire package of interventions, which might make it hard to distinguish which intervention was most effective regarding improved cancer rehabilitation care (with indicator adherence as effect measure). During the evaluation, we will try to get insight into the effect of each separate intervention by acquiring information from participants concerning the exposure to, use of and experiences with the different interventions.

A dilution of treatment effect could occur if patients participate in a PCRP themselves or when professionals and organizations use their own implementation strategies to achieve proper implementation. Local initiatives to optimize cancer rehabilitation during the intervention period might bias our study results. Therefore, information on local, non-study-related interventions will be additionally acquired during the process evaluation.

Hospitals, which agree to participate in our study, are probably committed to improve their quality of cancer rehabilitation care and the implementation of PCRPs. Their results will be generalizable for hospitals motivated to implement PCRPs. For hospitals less committed to achieve this goal, the outcomes might be less generalizable.

## Conclusion

Our study is intended to highlight the important role of the right implementation strategy to provide evidence-based PCRPs to the patients. We believe that strategies tailored to the current setting and barriers and facilitators found will optimize the success of implementation of PCRPs. If one or both implementation strategies prove to be effective, the knowledge about the effectiveness, feasibility and cost of the tailored implementation strategies can help to apply these PCRPs successfully in the Dutch and other healthcare systems. Additionally, our study might give insight in the additional effects of strategies directed at patients, professionals and organizations in an implementation strategy for PCRPs. Finally, if the implementation strategies are effective, this will lead to improved survivorship care leading to better quality of life for cancer survivors.

## Abbreviations

A-CaRe: Alpe d’HuZes Cancer Rehabilitation
EPOC: Effective Practice and Organisation of Care
ICER: incremental cost-effectiveness ratio
ICT: information and communication technology
MF: multi-faceted
PAM: Patient Activation Measure
PCRP: physical cancer rehabilitation programme
PD: patient-directed
QLQ-C30: European Organisation for Research and Treatment of Cancer Quality of life Questionnaire-C30
QoL: quality of life
